# A New Target Detection Method of Ferrography Wear Particle Images Based on ECAM-YOLOv5-BiFPN Network

**DOI:** 10.3390/s23146477

**Published:** 2023-07-18

**Authors:** Lei He, Haijun Wei, Qixuan Wang

**Affiliations:** Merchant Marine College, Shanghai Maritime University, Shanghai 201306, China; helei0014@stu.shmtu.edu.cn (L.H.);

**Keywords:** wear particle, image recognition, YOLOV5s, ECAM, weighted BiFPN

## Abstract

For mechanical equipment, the wear particle in the lubrication system during equipment operation can reflect the lubrication condition, wear mechanism, and severity of wear between equipment friction pairs. To solve the problems of false detection and missed detection of small, dense, and overlapping wear particles in the current ferrography wear particle detection model in a complex oil background environment, a new ferrography wear particle detection network, EYBNet, is proposed. Firstly, the MSRCR algorithm is used to enhance the contrast of wear particle images and reduce the interference of complex lubricant backgrounds. Secondly, under the framework of YOLOv5s, the accuracy of network detection is improved by introducing DWConv and the accuracy of the entire network is improved by optimizing the loss function of the detection network. Then, by adding an ECAM to the backbone network of YOLOv5s, the saliency of wear particles in the images is enhanced, and the feature expression ability of wear particles in the detection network is enhanced. Finally, the path aggregation network structure in YOLOv5s is replaced with a weighted BiFPN structure to achieve efficient bidirectional cross-scale connections and weighted feature fusion. The experimental results show that the average accuracy is increased by 4.46%, up to 91.3%, compared with YOLOv5s, and the detection speed is 50.5FPS.

## 1. Introduction

During the operation of mechanical equipment, friction occurs between parts and components, resulting in wear particles. Wear particles can directly reflect the operating status of mechanical equipment. Therefore, by detecting and identifying the number and type of wear particles, the running status of mechanical equipment can be monitored and warnings can be issued. Ferrography wear particle analysis technology is a method of detecting and identifying wear particles. It can judge the lubrication condition, wear mechanism, and severity of friction pairs through the color, quantity, size, shape, and texture of wear particles [1]. Traditional ferrography wear particle analysis requires manual detection. Due to the wide variety of wear particle characteristics and different shapes, the accuracy of manual detection is low, very cumbersome, time-consuming, and limited by professional knowledge. Therefore, it is of great significance to study the automatic detection and identification of wear particles. 

Many scholars have studied this. Peng et al. proposed an automatic wear particle recognition system based on AlexNet, which achieved a high accuracy rate with fewer training samples [2]. Identifying wear particle chains and overlapping wear particles was a challenge. Fan et al. proposed a new wear particle recognition network named FFWR-Net, which made the extracted features more representative and comprehensive, and improved the classification accuracy [3]. However, the model has no detection capability, and is essentially just an “image classifier”. Wang et al. proposed a two-level convolutional neural network model fused with BP and CNN [4]. This model realized the identification and automatic classification of wear particles. However, the recognition image features of wear particles were more obvious, and the types were fewer and were easier to distinguish. Peng et al. proposed a three-level classification convolutional neural network model that combined Inception-v3 and ANN for the classification of common fatigue, oxidation, and spherical wear particles [5]. This model was superior to the SVM-ANN classification algorithm, but it could not detect objects. In the face of multi-wear images, image segmentation is needed in advance. Xie et al. proposed a multi-channel coded convolutional neural network model called MCECNN, which can improve the visual clarity of images, improve the visible edges and surface features of images, and enhance the accuracy and generality of the image in the process of deep learning [6,7]. However, the model could not also detect objects. Qiu et al. proposed a ferrography wear particle image recognition technology based on a support vector machine, which had high detection accuracy but could not identify small wear particles, fatigue, or severe sliding wear particles [8,9].

At present, many scholars have introduced deep learning technology for wear particle image target detection, carried out a large amount of research, and achieved certain research results. Representative algorithms for target detection based on deep learning include Fast R-CNN [10], Faster R-CNN [11,12], Mask R-CNN [13], SSD [14], UIU-Net network [15], and YOLO series [16,17,18]. He et al. proposed a wear particle recognition algorithm based on Faster R-CNN, which can automatically extract the features of ferrography images, determine the type of wear particle, and count the number of various types of wear particles, but cannot identify overlapping wear particles and ignores small wear particles in the image [19]. Yang et al. proposed a wear particle digital characterization method based on Mask R-CNN, which can realize the identification of multiple target wear particles in a single image [20]. However, the accuracy and recall were only 44.5% and 56.6%, respectively. An Chao used the Mask R-CNN model to train an automatic wear particle recognition system, which was conducive to the detection of multi-scale wear particles and small target wear particles [21]. However, the training and recognition speed was slow, and the AP recognition was only 82%. Zhang et al. proposed two improved models based on the YOLO algorithm [22]. While ensuring the detection speed, the recognition rate of similar wear particles was improved, and the missed detection rate of small wear particles was reduced. However, the recognized mAP is only 85.37% and the detection speed is only 17 FPS. Peng et al. proposed a WP-DRNet network model based on YOLOv3 for automatic wear particle detection and classification, which can detect and identify multi-category wear particle images; however, it is only 87.2 percent accurate [23]. The detection ability of close or occluded targets and small targets was relatively weak. Wang et al. proposed a target detection algorithm based on the improved YOLOv4 [24]. Under the premise of ensuring accuracy, the number of network parameters decreased significantly and the recognition speed improved. However, the types of wear particles that are recognized are fewer, and images with small wear particles and complex backgrounds cannot be recognized. Faster R-CNN divides target detection into two stages, namely candidate region generation and region classification regression, and the accuracy rate is improved. However, compared with YOLOv5, Faster R-CNN is more computationally intensive and slower. Mask R-CNN is an instance segmentation algorithm; the model is easy to implement and training adds a small amount of computational overhead, and it achieves fast segmentation and recognition. However, compared with YOLOv5, the Mask R-CNN model has systematic errors and false edges when identifying overlapping targets. UIU-Net is a new network for small object detection, which embeds tiny U-Net into a larger U-Net backbone network to achieve multi-level and multi-scale feature representation of objects. However, compared with the YOLOv5 model, the UIU-Net model training is very slow, and it also generates redundancy, resulting in overfitting, positioning accuracy, and background information [25]. In summary, this paper selects the YOLOv5s target detection algorithm as a pre-improvement target detection algorithm [26]. Compared with other detection methods, this algorithm has great advantages in accuracy and speed and has strong real-time performance. However, there are deficiencies in the detection and identification of wear particles:

- The background of the ferrography image is complex and full of small wear particles, the edges are blurred, the surface texture is unclear, and the targets easily overlap;
- In the process of network feature extraction, it is easy to lose small target features;
- Overfitting may occur during model training.

In response to the above problems, this paper proposes a self-designed ferrography wear particle detection network, EYBNet, based on YOLOv5s, that can achieve better results in the detection of small, dense, and overlapping wear particles. The contributions of this paper are as follows:

- The MSRCR algorithm is used to process the image of ferrography wear particles to be detected to reduce the interference of the complex background of lubricating oil and improve the image contrast, thus improving the quality of the data set. Based on YOLOv5s and the channel information attention module (ECAM), an improved YOLOv5s network that fuses channel information is proposed, and a three-layer channel information attention module is added to the backbone of the feature extraction network of YOLOv5s. This improves the salience of small wear particles in the picture, enhances the feature expression ability of wear particles in the detection network, and realizes the accurate identification of small, dense, and overlapping wear particles.
- The original feature pyramid module in the feature fusion module of YOLOv5s is replaced with a weighted bidirectional feature pyramid (BiFPN) network structure to achieve efficient bidirectional cross-scale connection and weighted feature fusion, to obtain more feature information and thereby avoid the loss of wear particle feature information.
- DWConv is introduced to divide the convolution operation into two steps: depth convolution and point-by-point convolution. Depth convolution only performs convolution in the channel dimension, and only needs to learn channel-related parameters, and point-by-point convolution only performs convolution in the space dimension, and only need to learns spatially related parameters. This can greatly reduce the number of calculations and parameters, and improve the running speed, training speed, and generalization ability of the network.
- By optimizing the loss function, the learning efficiency of the model is improved, the weight of easy-to-classify samples is reduced, the anchor frame positioning is more accurate, the robustness of the model is enhanced, the identification is more accurate, and the overfitting phenomenon in training is reduced or avoided.

## 2. Method

### 2.1. Improved YOLOv5s Network Structure

**Figure 1:**
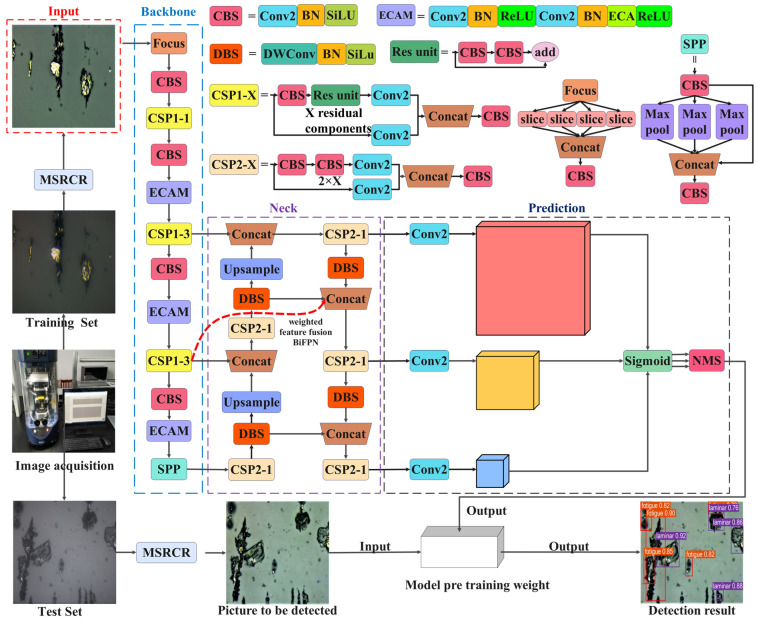
Structural framework of EYBNet.

Aiming at the problems of existing deep learning target detection models, namely that they are prone to false detection and missed detection of small target wear particles, dense wear particles, and overlapping wear particles, EYBNet mainly consists of four parts, namely Input, Backbone, Neck, and Prediction, as shown in Figure 1.

Input uses the Mosaic data enhancement method to randomly stack, zoom, crop, and arrange different images for splicing, and adopts the adaptive anchor frame calculation method, which automatically learns the size of the anchor frame according to the new data labels.

Adding ECAM to Backbone’s CBS module allows the module to operate with almost no additional computational complexity, improving model performance, improving data imbalance issues, reducing model parameters, improving model stability and flexibility, and helping to improve the accuracy and efficiency of wear particle identification. 

By improving based on Neck’s PAN, a new feature fusion path-weighted bidirectional feature pyramid network structure, BiFPN, is constructed, repeating the same layer multiple times for fusion to achieve higher-level feature fusion and improve the detection performance of the network for small targets. 

The Prediction part uses an improved loss function consisting of three parts: classification loss function, position loss function, and confidence loss function. In the post-processing of target detection, non-maximum suppression (NMS) is used to obtain the local maximum.

### 2.2. Image Enhancement

The collected wear particle images not only have complex features, but also may have problems such as blurred image details, poor contrast, and a large amount of noise due to external environmental interference, uneven light, and low pixel pixels in actual working environments. Image enhancement can effectively improve the definition of wear particle features in the image without significantly affecting the clarity speed, which is conducive to improving the accuracy of the model. In this paper, adaptive histogram equalization [27], a homomorphic filtering algorithm, and MSRCR [28] are used to enhance image features.

**Figure 2:**
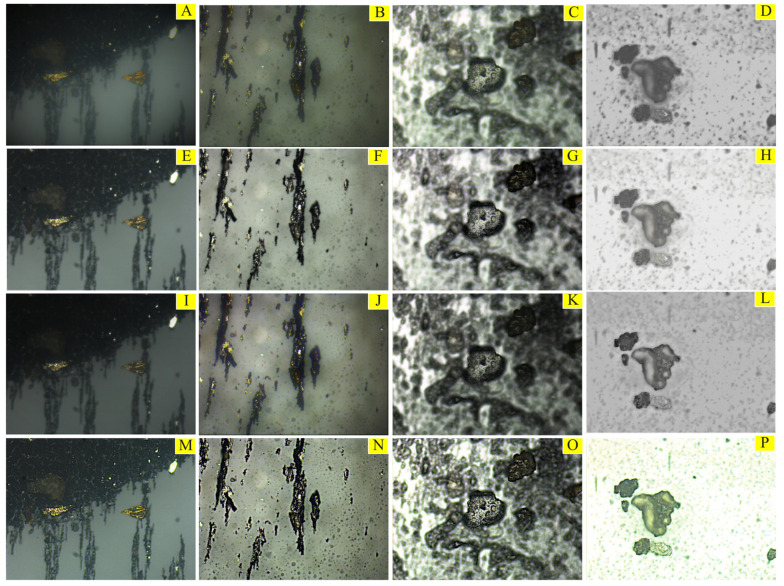
Effect comparison of different image feature enhancement algorithms: (**A**–**D**) the original input images; (**E**–**H**) the images processed by CLAHE; (**I**–**L**) the images processed by homomorphic filtering; (**M**–**P**) the images processed by MSRCR.

The image feature enhancement effects of each algorithm are shown in Figure 2, and Figure 2A–D are the original input images. Figure 2E–H are the images processed by CLAHE. The contrast has been significantly improved, but the problem of color deviation is severe, the image is darker, and the image details are not enhanced. Figure 2I–L are images processed by homomorphic filtering; some features become prominent and the contrast is locally improved, but the overall image is dark. Figure 2M–P are images processed by MSRCR, and the overall brightness and clarity are significantly improved, and the wear particle outline and surface texture are clearer without obvious noise problems. Then, the original image and the images processed by the three algorithms are input into EYBNet for detection. The experimental results show that the recognition accuracy of the original image is 90.5%, that of CLAHE is 91.6%, that of the homomorphic filtering algorithm is 92.2%, and that of MSRCR is 94.2%. In this paper, the MSRCR algorithm is selected for image feature enhancement, and the effect is the best.

### 2.3. Introduce an Efficient Channel Attention Module

**Figure 3:**
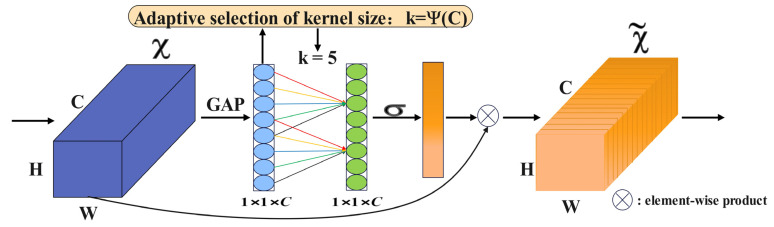
The principle of ECAM.

In the detection of ferrographic wear particle images, problems such as the complex background, missed detection of small targets, and false detection of overlapping wear particles are often encountered. To solve these issues, this paper introduces the ECAM attention module, a technique for enhancing the receptive field and inter-channel interactions of neural networks. The specific purpose of adding the ECAM attention module in the improved YOLOv5s is to improve the performance and accuracy of ferrographic wear particle image detection [29]. ECAM can enhance important feature representations, facilitate cross-channel interactions, and provide network adaptability. Thus, the detection results are improved and the network can better process images of different types of wear particles. The working principle is shown in Figure 3.

This module learns the channel attention through the parameter matrix $W_{a}$, as shown in Equation (1):

(1) $$W_{a}=\left[\begin{array}{cccccccc} w^{1,1} & \cdots & w^{1,a} & 0 & 0 & \cdots & \cdots & 0\\ 0 & w^{2,2} & 0 & w^{2,a+1} & 0 & \cdots & \cdots & 0\\ \vdots & \vdots & \vdots & \vdots & \ddots & \vdots & \vdots & \vdots \\ 0 & \cdots & 0 & 0 & \cdots & w^{C,C-(a+1)} & \cdots & w^{C,C} \end{array}\right],$$

where *C* is the number of channels and $w^{a,a}$ is the interaction between channels. This matrix form considers the interaction between different channels, avoiding the complete independence of channels. To improve the efficiency of information exchange, this paper only considers the correlation between a channel and its neighboring m channels, and gives channel weights as shown in Equation (2):

(2) $$w_{i}=\sigma \left(\sum_{j=1}^{m}w_{{}_{i}}^{j}y_{{}_{i}}^{j}\right),\;y_{{}_{i}}^{j}\in \Omega_{i}^{m},$$

where $\Omega_{i}^{m}$ represents the set of m channels adjacent to the channel $y_{i}$. The module utilizes a kernel size of *k* one-dimensional convolutions to realize parameter sharing of weight information and further improve the model performance, as shown in Equation (3):

(3) $$w=\sigma \left(C1D_{n}\left(y\right)\right),$$

where *C*1*D* stands for one-dimensional convolution and σ is a sigmoid function. Among them, the kernel size *k* is related to the channel, and there is a nonlinear functional relationship between dimension *C*, as shown in Equation (4):

(4) $$C=\phi \left(k\right)=2^{\left(\gamma \times k-b\right)},$$

The value of parameter *k* can be adaptively determined by Equation (5) as follows:

(5) $$k=\psi (C)={\left|\frac{\log_{2}(C)}{\gamma }+\frac{b}{\gamma }\right|}_{odd},$$

where ${\left|\cdot \right|}_{odd}$ means to take the nearest odd value; γ=2, b=1.

**Figure 4:**
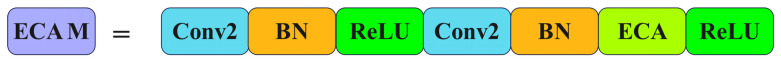
Structure of the channel information attention module.

ECAM is based on ECA and combines Conv2d, BatchNorm2d, and ReLU. The composition structure of the channel information attention module is shown in Figure 4.

Although the high-efficiency channel attention module can enhance the feature expression, the number and position of modules will affect the final experimental effect. Several experiments show that adding too many ECAMs or placing them too far ahead will weaken the feature extraction capability of the network to varying degrees and increase the difficulty of feature extraction. Therefore, this paper chooses to add three ECAMs to the backbone network.

### 2.4. Improvement of Feature Pyramid Structure

**Figure 5:**
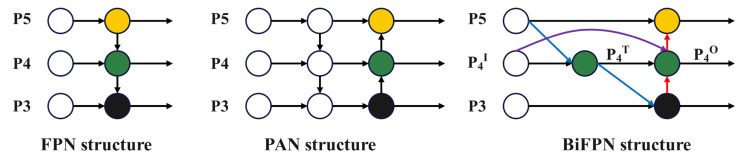
FPN, PAN, and BiFPN structure.

FPN is a fundamental component for recognizing objects of different sizes, but its traditional top-down structure is affected by one-way information flow restriction. To solve this problem, the adopted path aggregation network (PAN) adds a bottom-up aggregation path [30], which has higher accuracy, but it will also lead to an increase in parameter and calculational complexity. Therefore, this paper chooses weighted bidirectional (top-down + bottom-up) BiFPN [31]. The idea of BiFPN is to achieve path enhancement through efficient bidirectional cross-scale connection and weighted feature fusion. Firstly, top-down feature fusion is performed, and then bottom-up feature fusion is performed. The structure is shown in Figure 5.

The BiFPN structure is based on PAN, removing nodes with only one input, and adding a new aggregation path between the original input node and the output node. Through multiple superpositions, more feature information is fused. The BiFPN network uses the fast normalized fusion method for fusion with weights, as shown in Formula (6):

(6) $$O=\sum_{i}\frac{\omega_{i}I_{i}}{\varepsilon +\sum_{j}\omega_{j}},$$

where $I_{i}$ is the input feature, O is the output feature, $\omega_{i}$ and $\omega_{j}$ are the learnable weights, the ReLU activation function is used to scale the learnable weights between [0, 1], and ε=0.0001 is a small amount to ensure stable output.

Taking node P4 as an example, the two fusion feature processes are formed as follows:

(7) $$P_{4}^{T}=C\mathrm{onv}(\frac{\omega_{3}\cdot P_{4}^{I}+DBS(P_{5}^{I})}{\omega_{3}+\omega_{4}+\varepsilon }),$$

(8) $$P_{4}^{O}=C\mathrm{onv}(\frac{\omega_{3}\cdot P_{4}^{I}+\omega_{4}\cdot P_{4}^{T}+\omega_{4}\cdot DBS(P_{3}^{O})}{\omega_{3}+\omega_{4}+\omega_{5}+\varepsilon }),$$

**Figure 6:**
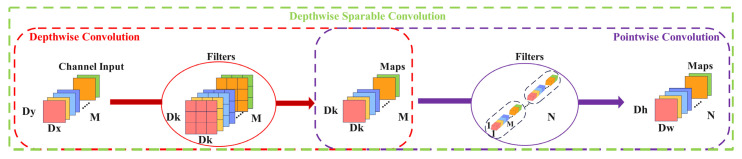
Depthwise separable convolution structure.

In Equations (7) and (8), $P_{4}^{T}$ is the top-down intermediate feature (the middle green circle in Figure 6), $P_{4}^{OUT}$ is the output feature from bottom to top (the rightmost green circle in Figure 6), Conv represents the convolution operation, $\omega_{i}\geq 0$ is the learnable weight, and DBS represents the up-sampling or down-sampling operation of the input. The remaining features are constructed similarly.

To ensure the network has higher accuracy and to reduce the influence of introducing a feature fusion pyramid on network detection speed, the convolution block in the CBS module in Neck is replaced by depthwise separable convolution (DWConv), which can overcome the problem that the high calculation amount caused by traditional convolution cannot improve the running speed. At the same time, the parameters are reduced, the model size is reduced, and the calculation speed is improved [32]. The depthwise separable convolution structure is shown in Figure 6.

### 2.5. Improved Loss Function

The loss function of the network reflects the difference between the prediction value and the real values. For network parameter updates, it provides a reliable basis. The smaller the loss function value, the better the model’s robustness [33].

The composition of YOLOv5s’ loss function is shown in Equation (9):

(9) $$L=L_{GIOU}+L_{BCE}+L_{BCE},$$

In the equation, $L_{GIOU}$ is GIOU loss and $L_{BCE}$ is BCE loss.

Among them, the GIOU loss function is shown in the following (10):

(10) $$L_{GIOU}=1-IOU+\frac{\left|C-B\cup B^{gt}\right|}{\left|C\right|},$$

where *IOU* is the intersection ratio between the prediction frame and the real frame, $B=\left\{x,\;y,\;\omega ,\;h\right\}$ is the size of the prediction frame, $B^{gt}=\left\{x^{gt},\;y^{gt},\;\omega^{gt},\;h^{gt}\right\}$ is the size of the real frame, and *C* is the minimum area of *B* and $B^{gt}$. The further the distance between the real frame and the prediction frame, the greater the value of *C*, and the greater the value of *C* minus the area of the prediction frame and the real frame, the more it finally tends to 1. Although *GIoU* solves the gradient problem of *IOU*, *GIoU* is unstable and converges slowly.

The BCE loss function is shown in Equation (11):

(11) $$L_{BCE}=-\sum_{\left(i,j\right)}\left[G(i,j)\log(T(i,j))+(1-G(i,j))\log(1-T(i,j))\right],$$

where G(i,j) represents the label of pixel (i,j), and T(i,j) is the predicted probability value of pixel (i,j) being tampered with.

The improve total loss function $L_{toal}$ in this paper consists of three parts: improved bounding box loss $L_{EIOU}$, the improved classification loss $L_{QFocal}$, and the improved confidence loss $L_{BCE}$, as shown in Equation (12):

(12) $$L_{toal}=\lambda_{EIOU}L_{EIOU}+\lambda_{QFocal}L_{QFocal}+\lambda_{BCE}L_{BCE},$$

where $\lambda_{EIOU}$, $\lambda_{QFocal}$, and $\lambda_{BCE}$ are three hyper-parameters with corresponding losses, which are set to 1.00, 0.50, and 0.05 in this paper. $L_{EIOU}$ is EIOU loss, $L_{QFocal}$ is quality focal loss, and $L_{BCE}$ is BCE loss.

Focal loss is a loss function used to solve the problem of class imbalance in object detection tasks [34]. In the ferrographic wear particle image, there may be some wear particles that are difficult to classify, such as those with a fuzzy shape or small size, or similar background noise. These difficult-to-classify wear particle samples are critical to the accuracy of wear particle detection. $L_{QFocal}$ increases the weight of hard-to-classify wear particles by adjusting the weight of the loss function, so that the network can pay more attention to these hard-to-classify wear particles and improve their detection ability, as shown in Equation (13):

(13) $$L_{QFocal}=-{\left|y-\sigma \right|}^{\beta }\left[\left(1-y)\log(1-\sigma )+y\log(\sigma \right)\right],$$

In the equation, y is a continuous sample label with a value between 0 and 1, and σ is the predicted value of the model; ${\left|y-\sigma \right|}^{\beta }$ is an adjustment factor for controlling the weight by using the parameter β. When the real value is close to the predicted value, β is smaller; otherwise, it tends to 0.

By introducing additional compensation items, EIOU loss can more accurately measure the degree of overlap between abrasive grain target frames, thereby improving the accuracy of matching. It is also more sensitive to changes in the position and scale of target frames, can better adapt to various changes in the shape and scale of wear particles, and support end-to-end object detection network training, as shown in Equation (14):

(14) $$L_{EIOU}=1-IOU+\frac{\rho^{2}\left(b,b_{gt}\right)}{C_{b}{}^{2}}+\frac{\rho^{2}\left(w,w_{gt}\right)}{C_{w}^{2}}+\frac{\rho^{2}\left(h,h_{gt}\right)}{C_{h}^{2}},$$

where ρ represents the Euclidean distance between two center points, b and $b_{gt}$; ω and $w_{gt}$, and *h* and $h_{gt}$, respectively, represent the center point, width, and height of the prediction frame and the real frame; $C_{b}$, $C_{w}$, and $C_{h}$ are the diagonal length, width, and height of the minimum bounding rectangle of the prediction frame and the real frame, respectively.

## 3. Experiment 

### 3.1. The Overall Process of this Experiment

**Figure 7:**
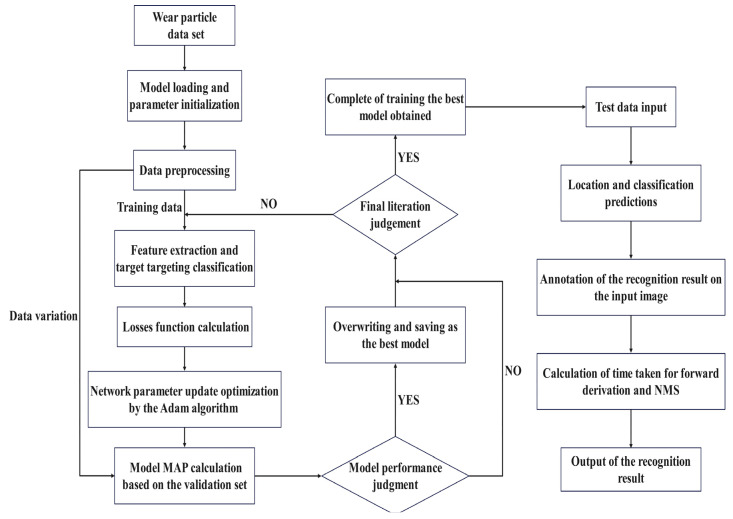
Overall flow chart of the experiment.

The goal of training the network model is to continuously adjust the parameters in the network to maximize the score of the correct category and minimize the score of the incorrect category. The difference between the ideal correct score and the score calculated by the model is called the loss. Therefore, the ultimate goal of the training model is to find a set of weights that maximizes the average loss on the training set. The overall flow chart of this experiment is shown in Figure 7.

### 3.2. Collection and Production of Data Sets

**Figure 8:**
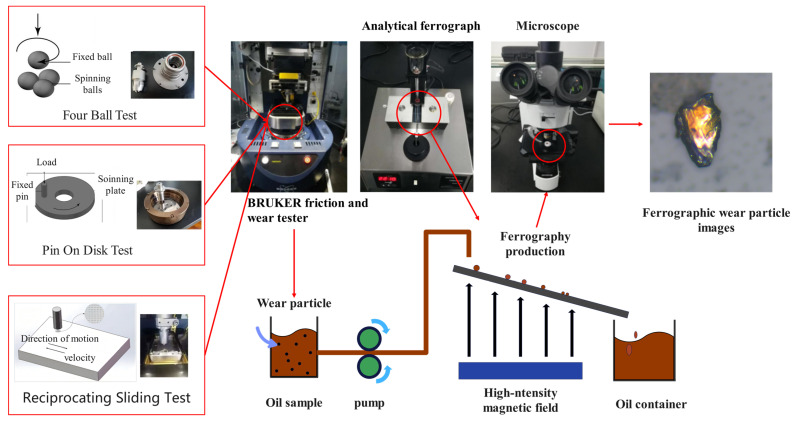
Bruker’s UMT universal mechanical tester.

The data set used in this paper was passed through Bruker’s UMT universal mechanical tester to produce different working conditions and different types of wear particles required by the experiment. As shown in Figure 8, three tribological tests were carried out considering different wear patterns: the pin-on-disk test, pin-on-plate test, and four-ball test. The pin-on-disk test was conducted in laboratory air at a temperature of 22 °C and relative humidity of 50%, under a load of 30 kg (294 N), and at a rotational speed of 900 r/m for 25 h. The pin-plate test used a reciprocating module instead of a rotating module and operated under a load of 48 kg (470.4 N) for 12 h. The maximum load and speed of the four-ball test were set to 1500 N and 300 r/min, and the running time was set to 30 h.

**Figure 9:**
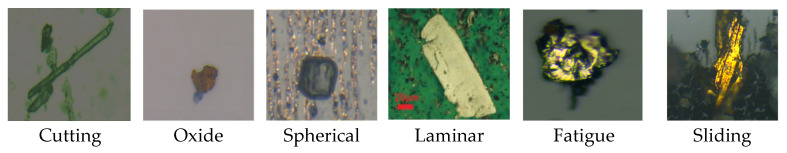
Images of wear particles with different characteristics and wear types.

After a series of experiments, the experimental conditions for the formation of various wear particles were finally established. Note that there may be several different classes of wear particles within a single ferrography wear particle image. After that, it is necessary to preprocess the collected ferrography wear particle images, and label them with labeling software. After labeling, .txt is used as the suffix, the size of the image is 640 × 640, and the file name is consistent with the picture. The data set is classified into six types of wear particles: Cutting, Oxide, Spherical, Laminar, Fatigue, and Sliding, among which Laminar, Fatigue, and Sliding wear particles are highly similar and difficult to distinguish, as shown in Figure 9. When training the model, the labeled data set is divided into a training set, validation set, and test set. The training set is used to train the model, the validation set is used for parameter tuning and evaluation of the model, and the testing set is used to evaluate the generalization ability of the model [35].

In this paper, a total of 5440 wear particles were marked and divided into training set: validation set: test set in a ratio of 6:2:2. The training set, validation set, and test set contained 3264, 1088, and 1088 wear particles, respectively, as shown in Table 1.

### 3.3. Experimental Environment and Parameter Settings

The software and hardware platform configuration parameters of this experiment are shown in Table 2 and the initial parameter settings are shown in Table 3.

### 3.4. Evaluation Index

In order to evaluate the performance of the model, the precision (*P*), the recall (*R*), the average precision (*AP*), the mean average precision (*mAP*), and the harmonic mean, $F_{1}$, of the precision and recall, were the main evaluation indexes used; the specific expressions are as follows:

(15) $$P=\frac{TP}{TP+FP},$$

(16) $$R=\frac{TP}{TP+FN},$$

(17) $$F_{1}=\frac{2}{\frac{1}{P}+\frac{1}{R}}=2\frac{PR}{P+R},$$

(18) $$AP=\sum_{i=1}^{n-1}\left(r_{i+1}-r_{i}\right)p(r_{i+1}),$$

(19) $$mAP=\frac{\sum_{j=0}^{m}AP(j)}{m},$$

This paper sets the correct intersection to union ratio (IOU≥0.5), which reflects the degree of overlap between the predicted bounding box and the real bounding box. TP indicates correctly detected wear particles, FP indicates incorrectly identified wear particles, and FN indicates unrecognized wear particles; m represents the number of samples in the test set and mAP@0.5 represents the average precision when the IOU threshold is set to 0.5.

The model speed evaluation index uses FPS (frames per second), which represents the number of frames per second of the screen transmission, and the equation is as follows:

(20) $$FPS=\frac{1}{t_{average}},$$

where $t_{average}$ is the average time required to detect a picture. The larger the FPS, the faster the model inference speed.

## 4. Experimental Results and Analysis

### 4.1. Analysis of Experimental Results

**Figure 10:**
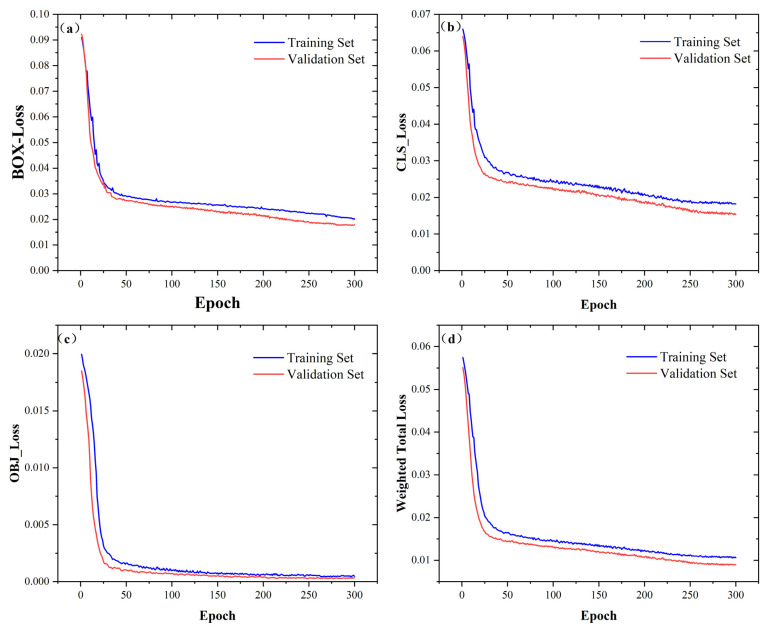
Variation of loss function curve: (**a**) BOX-Loss change curve; (**b**) CLS-Loss change curve; (**c**) OBJ-Loss change curve; (**d**) Weighted Total Loss change curve.

This paper trains on the data set according to the model configuration in Section 3.3. The loss function changes on the training set and the validation set are recorded, as shown in Figure 10. The loss function includes BOX-Loss, CLS-Loss, OBJ-Loss, and Weighted Total Loss.

It can be seen from Figure 10 that all loss functions show a downward trend, indicating that the model is not overfitting during continuous learning. Through the evaluation of the validation set, an optimal set of hyperparameter combinations can be selected to minimize the average loss of the model. The total loss on the training set finally converges to around 0.012, while the total loss on the validation set ultimately converges to around 0.008.

**Figure 11:**
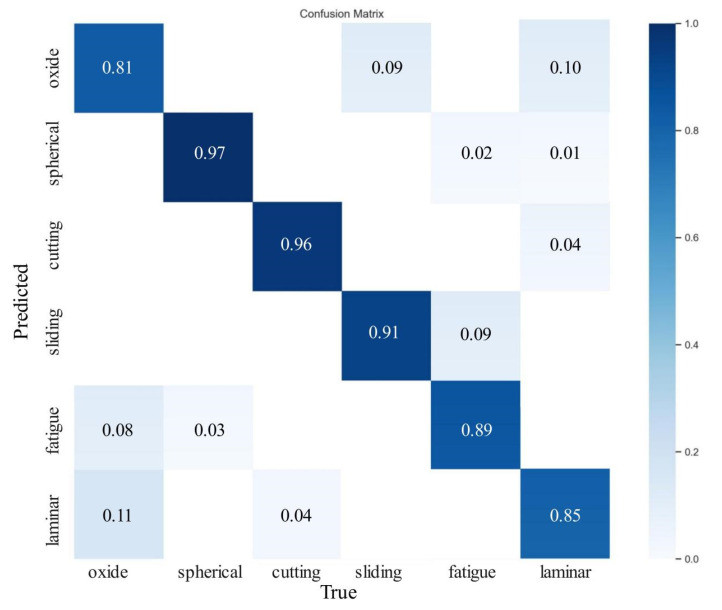
Confusion matrix of six wear particles.

In this paper, the method of transfer learning is used to speed up the model training, and the weight of the best performance model trained on the data set is used as the pre-training weight of EYBNet [36,37]. During the training process, the number of iterations was set to 300 rounds. The initial learning rate was 0.01, the learning rate of the neural network was adjusted in the way of OneCysleLR, and the learning rate finally attenuated to 1 × 10^−4^. The test set data was inputted for final evaluation, and recognition results of six kinds of wear particles were obtained, as shown in Table 4. To ensure the generalization ability and performance of the model, the confusion matrix is shown in Figure 11.

**Figure 12:**
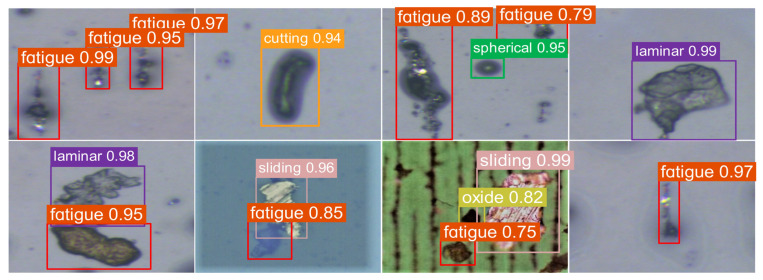
EYBNet visual results of detection and identification.

As shown in Table 4 and Figure 11, the accuracy of detection and recognition of six wear particles of EYBNet is more than 80%, with the highest accuracy rate for spherical wear particles, reaching 96.9%. Recall exceeded 70%, with the highest recall for identifying layered wear particles, reaching 85.5%. Through F1, it can be known that most of the test accuracy results of the model exceeded 80%. The experimental results show that EYBNet can complete the detection and recognition of six kinds of wear particles, with high precision and good generalization ability. However, the disadvantage is that Laminar and Fatigue wear particles are wear particles with high similarity. Their aspect ratios are not much different, and their boundary features are similar. Only the texture features are slightly different, which makes it difficult to distinguish the model, so the accuracy rate is low at 90%. In addition, since the color of oxidized wear particles is black, it is easy to mistakenly detect the other five black wear particles as oxidized wear particles, resulting in an accuracy rate of only 81%. In order to more intuitively assess the generalization ability of the model, this article shows some visual results of ferrographic wear particle image detection and recognition, as shown in Figure 12.

**Figure 13:**
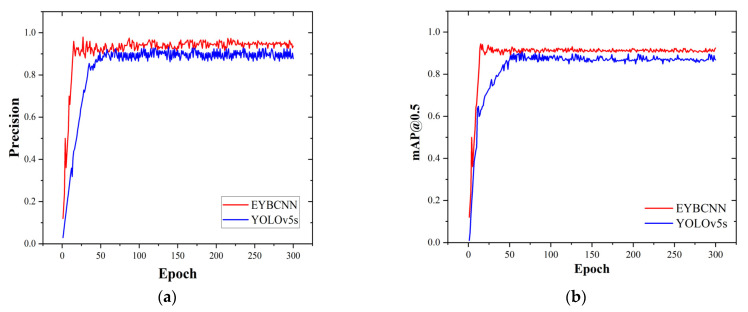
Change curve of evaluation index values: (**a**) precision change curve; (**b**) average precision change curve.

Comparing the algorithm of this paper with YOLOv5s, Figure 13 shows the specific trend of accuracy and mAP@0.5 indicators in the iterative process.

From Figure 13, it can be seen that the accuracy rate of YOLOv5s reached about 0.86 when the number of iteration rounds exceeded 35 rounds, and ultimately stabilized at around 0.896 as the number of iteration rounds increased. In the 15th round of EYBNet, the accuracy rate increased to about 0.96, and stabilized at about 0.942 as the number of iterations increased. Compared with the YOLOv5s model, EYBNet has a faster rate of convergence and higher accuracy. In addition, it can be seen that the average precision of YOLOv5s only reached about 0.89 when the number of iterations rounds exceeded 51, and finally stabilized at about 0.872 as the number of iterations increased. However, in the 15th round of EYBNet, the mean value of the average precision increased to around 0.94, and remained stable at about 0.913 as the number of iterations increased. Compared with YOLOv5s, EYBNet converges faster and has a higher average precision.

To sum up, given the problem that the current ferrography wear particle detection model has misdetection and missed detection of small, dense, overlapping wear particles, the model proposed in this paper can accurately identify various types of wear particles in complex oil background environments, and the algorithm is feasible. At the same time, the algorithm in this paper has a faster convergence speed and smaller loss, which can effectively solve the problems raised in this paper.

### 4.2. Ablation Experiment

#### 4.2.1. Ablation Experiments with Different Attention Mechanisms

To evaluate the improvement effect of the attention mechanism ECAM on YOLOv5s, YOLOv5s was improved based on the CBAM, CA, and SE attention mechanisms. With a training frequency set at 300 times, the performance of the above four attention mechanisms was then evaluated. The experiment results are shown in Table 5.

According to Table 5, it can be seen that the four attention mechanisms all have an improvement effect on YOLOv5s. Compared with CBAM, the accuracy of ECAM is 0.1% lower than that of CBAM; its recall, F1, and mAP are 0.2%, 0.1%, and 0.3% higher than those of CBAM, respectively; and the model occupies 1.1 MB less memory than CBAM. Compared with CA, the accuracy, recall, F1, and mAP of ECAM are 1.8%, 0.3%, 1.0% and 1.8% higher, respectively, than those of CA. The memory usage of the two models is similar, and the number of frames transmitted per second is 0.8 FPS less. In contrast, the accuracy of ECAM is 0.5% higher than that of SE, the recall is 0.1 percentage points lower, and F1 and mAP are 0.2% and 0.7% higher, respectively. From the above analysis, it can be seen that the comprehensive performance advantage of the ECAM attention mechanism is more obvious, and is more conducive to the accurate detection of multiple targets among small, similar, and overlapping wear particles.

#### 4.2.2. Improved Module Ablation Experiment

In order to more comprehensively analyze the superiority of various improvement modules in EYBNet for wear particle detection, this paper designs ablation experiments based on YOLOv5s, and analyzes the contribution of each improvement strategy to the network, as shown in Table 6. From the experiments, it is found that each module results in varying degrees of improvement in the overall performance of the model.

After adding the MSRCR algorithm to model 2, the local contrast of the image is improved, more image details are obtained, and image blur interference is reduced. Compared with the data of model 1, it is easy to find that the introduction of the MSRCR algorithm successfully improved the accuracy by 4.24%, the recall increased by 1.72%, the F1 increased by 2.93%, and the average precision increased by 1.83%. The detection speed of the two models is close.

Model 3 introduces ECAM into the backbone network, which enhances the weight ratio of the wear particle area in the image, highlights the wear particle features, and effectively solves the problem of difficult feature extraction caused by the complex background of the oil image, as well as the loss of network propagation feature information. After introducing the attention mechanism, it is easy to find that it successfully optimized the recognition performance of the network for small targets and multi-target wear particles. Compared to model 1, its accuracy is increased by 4.58%, the recall is increased by 7.74%, F1 is increased by 6.21%, average precision is increased by 4.69%, and speed is increased by 2.0 FPS.

After adding the BiFPN module to model 4, it has more positive significance in improving the accuracy of identifying fine wear particles and solving the problem of missed detection of wear particles. The overall accuracy rate increased by 2.12%, the recall increased by 6.51%, the F1 improved by 4.45%, and the average precision increased by 5.49%. This module creates a new path from bottom to top, treating each bidirectional path as a feature network layer, and repeating the same layer multiple times to achieve higher-level feature fusion, which is conducive to small target detection. This can greatly improve the accuracy of small, object standards, and at the same time increase the speed by 0.9 FPS. The problem of missed detection and wrong detection of tiny wear particles is solved, but for wear particles with slender and variable shapes and large annotation boxes, adding feature layers in the BiFPN module will lead to parameter redundancy, resulting in a lower accuracy improvement.

Model 5 replaced the Conv module of model 4 with the DWConv module. Compared with the data of module 4, the accuracy rate increased by 0.44%, the recall increased by 0.12%, the F1 increased by 0.22%, the average precision increased by 1.74%, and the speed increased by 2.5 FPS. It can be intuitively felt that the introduction of this module can reduce the amount of calculation, increase the speed, and obtain more feature information.

After changing the loss function to focal loss in model 6, the distance between the prediction frame and the target frame, the overlap rate, and the scale influence are considered, so that it can ensure the good convergence speed during the training process and it does not easily diverge. Based on the original network, the accuracy rate increased by 1%, the recall increased by 5.16%, the F1 increased by 3.14%, the average precision increased by 3.09%, and the speed increased by 1.63 FPS. After the improvement, the loss function identification has accurate identification and more accurate positioning, which can alleviate the data imbalance to a certain extent.

Through the data comparison of the ablation experiment, it was found that the performance improvement of model 7 after adding various improved modules, that is, the EYBNet proposed in this paper, was the most significant. The algorithm presented in this article has greatly improved the problem of detecting similar, overlapping, and fine wear particles. The six types of detection targets have achieved good detection results. Based on the original network, the accuracy rate increased by 5.13%, the recall increased by 6.02%, F1 increased by 5.63%, the average precision increased by 4.46%, and speed increased by 10.2 FPS. The effectiveness of the algorithm in this paper was verified for wear particle identification.

### 4.3. Performance Comparison of Different Algorithms

In order to verify the superiority of EYBNet, this paper compares and tests the performance of different networks on the same data set, and selects the commonly used models such as Faster-RCNN, SSD, and YOLO series for comparison. The experimental results are shown in Table 7.

From Table 7, it can be seen that the AP of each wear particle of EYBNet proposed in this paper is significantly higher than that in other models. The average precision of EYBNet is as high as 91.3%, which is 20.1%, 11.3%, 10.4%, 9.9%, and 3.9% higher than that in other models, respectively; and the F1 of EYBNet is as high as 90.1%, which is 20.5%, 12.5%, 10.3%, 9.9%, and 4.8% higher than that in other models, respectively. At the same time, the detection speed of EYBNet is also much higher than that of other models, reaching up to 50.5 FPS.

**Figure 14:**
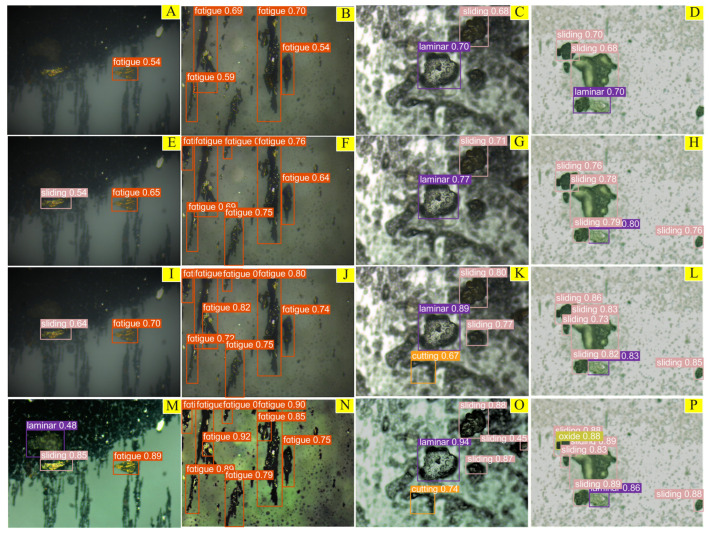
Comparison of detection and recognition results of different algorithms:(**A**–**D**) the SSD test results; (**E**–**H**) the Faster-RCNN test results; (**I**–**L**) the YOLOv5s test results; (**M**–**P**) the EYBNet test results.

To more directly and objectively evaluate the method in this paper, this paper selects wear particle images with small and scattered targets; wear particle images with many and similar targets; overlapping wear particle images with a complex background; and wear particle images that interfere with target detection and the blurred and aggregated target edge. These four different wear particle images were compared and tested, and the recognition results are shown in Figure 14. The numbers on the prediction box are the confidence level, which represents the certainty probability of the output result. Figure 14A–D show SSD test results; Figure 14E–H are Faster-RCNN test results; Figure 14I–L are YOLOv5s test results; and Figure 14M–P show EYBNet test results.

From Figure 14, it can be seen that for the SSD test results, there are missed detections of wear particles in Figure 14A, and there are missed detections of wear particles and overlapping wear particles in Figure 14B–D. In the case of false detection, the confidence scores of all results are at a low level of 0.54–0.70. Although Faster-RCNN recognizes most targets with a confidence level of 0.63–0.89, it easily misses detection of overlapping wear particles, such as shown in Figure 14H, in which a detection frame contains two different overlapping wear particles. YOLOv5s can identify most wear particles; however, when the target of the wear particle is small, the wear particles are close to each other and the features are occluded, or the background environment is complex and affected by a large amount of interference, YOLOv5s cannot accurately detect and identify wear particles, as shown in Figure 14J,K. In the complex black background, only EYBNet could recognize Laminar wear particles, and other algorithms did not recognize them. At the same time, the recognition confidence of similar Fatigue and Sliding abrasive particles was above 85%, as shown in Figure 14M,O. For the difficult-to-recognize Fatigue wear particles, compared with that of the other four algorithms, the confidence level in EYBNet was greatly improved, and there were no missed detections or false detections, as shown in Figure 14N. For the identification of small target wear particles and occluded overlapping wear particles, EYBNet also has higher confidence than the other four algorithms, and achieved accurate identification and classification without missing detection, as shown in Figure 14P.

## 5. Conclusions

This paper proposes the EYBNet wear particle detection network, presents detection experiments on ferrography wear particle images collected through a series of friction and wear experiments, and draws the following conclusions:

(1) Through different attention mechanism ablation experiments and improved module ablation experiments, it was verified that the improved methods of the algorithm presented in this paper are effective in identifying and detecting small, dense, and overlapping wear particles.
(2) Comparing EYBNet with SSD, Faster-RCNN, and YOLO series algorithms on the same experimental platform, the AP, F1, mAP, and recall of EYBNet for each type of wear particle detection are significantly higher than those of other models, successfully solving the problem of missed detection and false detection of wear particles.
(3) EYBNet has a faster detection speed while ensuring detection accuracy. Its detection speed reaches 50.5 FPS, which provides important conditions for the subsequent real-time online status analysis and fault diagnosis of mechanical equipment.

In future research, we will focus on online oil detection, gradually solve the problems encountered in the online detection of ferrography wear particles, and design a model with higher accuracy, a smaller model with faster speed and stronger generalization ability, and a deep learning model that is more suitable for online oil detection.

## Figures and Tables

**Table 1:** Number of samples in training, validation, and test sets.

Wear Debris	Cutting	Oxide	Spherical	Laminar	Fatigue	Sliding
Training Set	456	420	487	515	722	664
Validation Set	176	138	157	163	230	224
Test Set	200	135	153	154	220	226

**Table 2:** Software and hardware platform configuration parameters.

Configuration Name	Version Parameter
Operating system	Windows10
CPU	Intel(R) Core(TM) i9-10920X CPU @3.50 GHz,32 GB
Graphics card × 2	Nvidia RTX3090
Pytorch	1.8.1
CUDA	11.1

**Table 3:** Parameter settings.

Parameter Name	Parameter Values
Momentum	0.937
Batch size	32
Weight decay	0.0005
Learning rate	0.01
Epoch	300

**Table 4:** Wear particle identification results.

Type	TP	FP	FN	P/%	R/%	F1/%
Cutting	165	6	29	96.5	85.1	90.41
Oxide	90	21	24	81.1	78.9	80.00
Spherical	126	4	23	96.9	84.6	90.32
Laminar	115	20	19	85.2	85.8	85.50
Fatigue	142	18	60	88.8	70.0	77.78
Sliding	175	18	33	90.7	84.1	87.28

**Table 5:** Results of ablation experiments with different attention mechanisms.

Baseline Network	Attentional Mechanisms	P/%	R/%	F1/%	mAP@0.5/%	Memory Occupancy /MB	FPS
YOLOv5s	Nothing	89.6	81.4	85.3	87.4	15.6	40.3
	CBAM	93.8	87.5	90.5	91.2	15.1	41.9
	CA	91.9	87.4	89.6	89.7	14.1	41.5
	SE	93.2	87.8	90.4	90.8	14.2	42.0
	ECAM	93.7	87.7	90.6	91.5	14.0	42.3

**Table 6:** Results of ablation experiment with the improved module.

	Model	P/%	R/%	F1/%	mAP@0.5/%	FPS
1	YOLOv5s	89.6	81.4	85.3	87.4	40.3
2	YOLOv5s + MSRCR	93.4	82.8	87.8	89.0	40.5
3	YOLOv5s + ECAM	93.7	87.7	90.6	91.5	42.3
4	YOLOv5s + BiFPN	91.5	86.7	89.1	92.2	41.2
5	YOLOv5s + BiFPN + DWConv	91.9	86.8	89.3	93.8	42.8
6	YOLOv5s + Ltoal	90.5	85.6	88.0	90.1	41.9
7	YOLOv5s + ALL	94.2	86.3	90.1	91.3	50.5

**Table 7:** Detection and identification results of different network models.

Model	AP						mAP@0.5/%	F1/%	FPS
	Cutting	Oxide	Spherical	Laminar	Fatigue	Sliding			
SSD	86.5	70.9	85.3	65.1	70.9	72.5	71.2	69.6	10.2
Faster-RCNN	93.3	77.7	96.9	67.2	79.1	83.8	80.0	77.6	5.6
YOLOv3	94.5	74.5	96.7	73.2	79.2	85.3	80.9	79.8	16.9
YOLOv4	95.6	78.8	97.8	79.2	71.5	89.5	81.4	80.2	24.4
YOLOv5s	96.5	80.6	98.5	85.6	72.5	90.7	87.4	85.3	40.3
EYBNet	97.1	89.8	99.6	90.2	86.7	93.6	91.3	90.1	50.5

## Data Availability

The data that support the findings of this study are available from the corresponding author upon reasonable request.

## Abbreviations

The following abbreviations are used in this manuscript:

BiFPN	Bidirectional feature pyramid network
MSRCR	Multi-Scale Retinex with Color Restore
CLAHE	Contrast Limited Adaptive Histogram Equalization
ECAM	Efficient channel attention module
PAN	Path aggregation network
DWConv	Depthwise separable convolution
RCNN	Region convolutional neural network
SSD	Single-shot multibox detector
IoU	Intersection of Union
CBAM	Convolutional block attention module
YOLO	You Only Look Once
CNN	Convolutional neural network
GIoU	Generalized Intersection over Union
